# A case of cutaneous granuloma secondary to magico-religious practice: Magic, mysticism, and mercury

**DOI:** 10.1016/j.jdcr.2024.07.018

**Published:** 2024-08-05

**Authors:** Janice Jiang, Harold Winfield

**Affiliations:** Department of Dermatology, MetroHealth Medical Center, Case Western Reserve University, Cleveland, Ohio

**Keywords:** foreign body granuloma, mercury granuloma, subcutaneous mercury injection

## Introduction

Subcutaneous injection of mercury is rare and can be accidental or intentional. While some intentional exposures are related to psychiatric issues, self-harm and suicide attempts, the vast majority of intentional exposures are related to magico-religious practices. We report a case of intentional mercury injection for the purpose of “warding off evil” in an Indian Hindu woman resulting in a granulomatous tissue reaction 10 years after exposure.

## Case report

**Fig 1:**
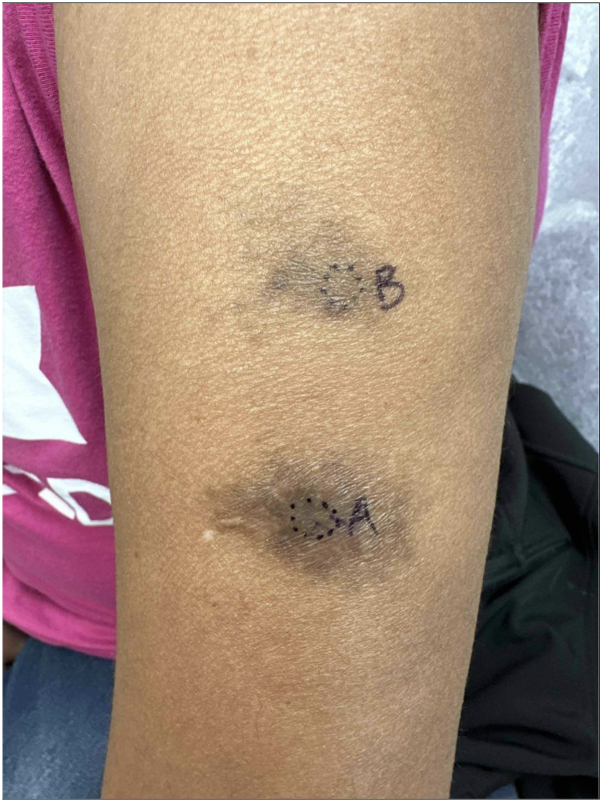
Poorly-demarcated indurated, asymmetric brown-tan plaques on the left arm.

**Fig 2:**
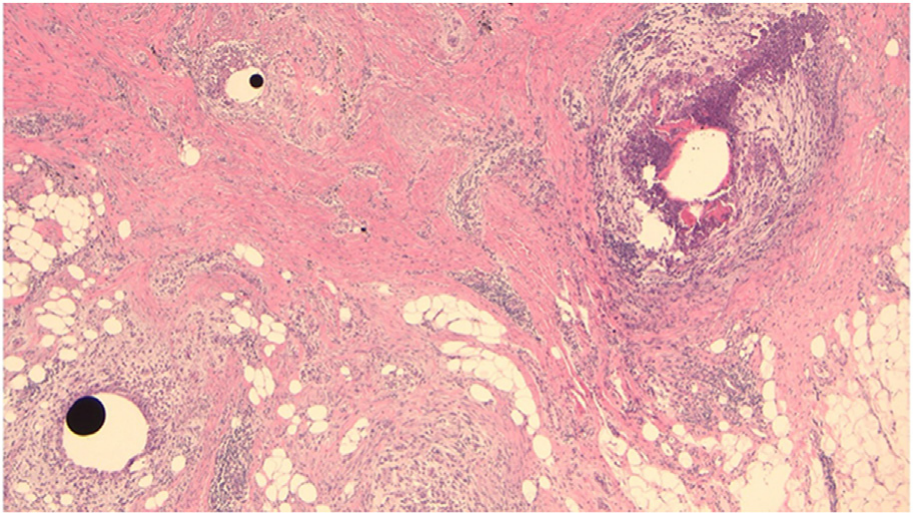
Multiple lacunae with round radio-opaque material, abscesses, scar, and exogenous pigment seen on low power (4×).

**Fig 3:**
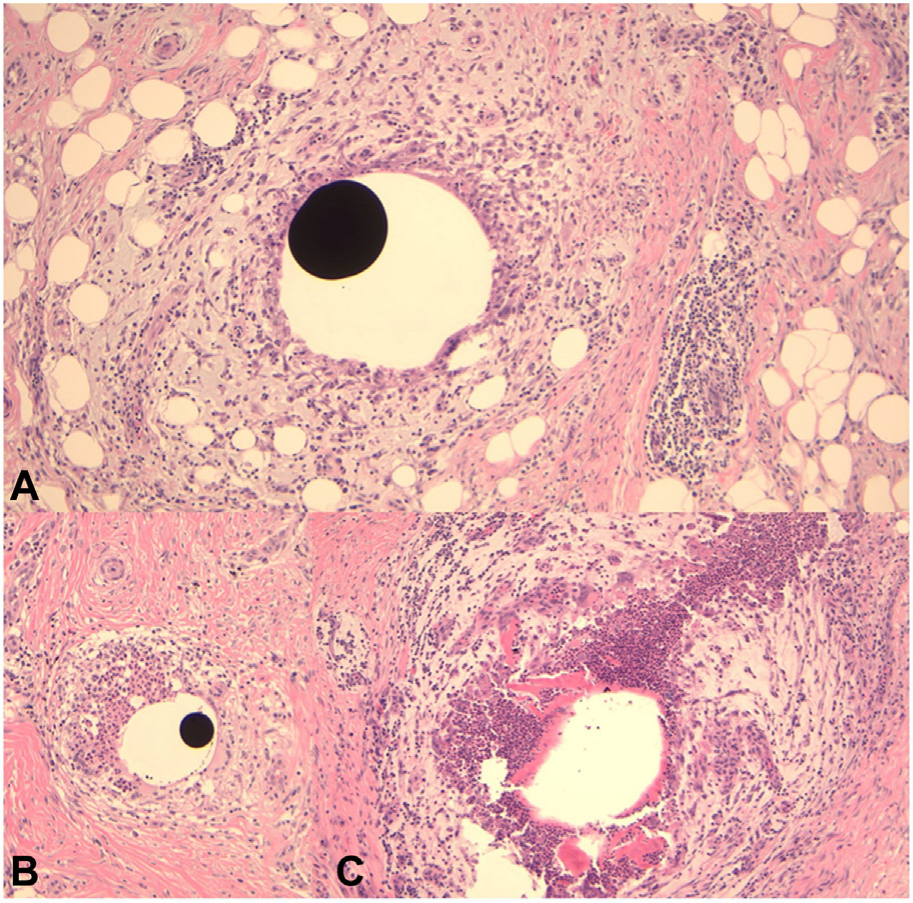
Lacunae with black radio-opaque material (**A** and **B**) and abscess comprised of neutrophils and eosinophils (**C**) can be seen at higher power (10×).

A 55-year-old Indian female presented to dermatology clinic with complaint of 2 irregular hyperpigmented plaques on the left upper arm (Fig 1). The patient initially denied any history of trauma or injections in the area. The patient reported itching in the affected areas but denied pain or any systemic symptoms such as fevers, chills, neurologic compromise, weight loss, or malaise. No neurologic deficits, alopecia, nail changes, or other physical exam findings were noted on initial evaluation. The differential diagnosis included morphea, dermatofibrosarcoma protuberans, extragenital lichen sclerosis, and sarcoidosis. Imaging studies were not considered at the time due to the lack of a clinical suspicion for implantation trauma. Two 4-mm punch biopsies were taken from the centers of each plaque. Histology showed broad zones of stromal scarring with well circumscribed areas of noncaseating granulomatous inflammation (Fig 2). Some areas demonstrated suppurative inflammation. There were admixed eosinophils. Within the granulomas were lacunae containing ovoid deposits of opaque black material, ranging in size from 0.02 to 0.1 mm in diameter, consistent with a metallic substance (Fig 3). Within the fibrotic stroma were aggregates of red hued crystalline debris measuring 0.01 to 0.02 mm in diameter. This material was thought to represent the formation of mercuric oxides.

After discussing the biopsy findings with the patient, she volunteered that 10 years prior to presentation, her father had given her a subcutaneous injection containing metallic mercury for the purpose of “warding off evil”. She also reported that she had at least 2 other family members with similar clinical findings due to mercury injections who declined to come in for medical treatment. The patient was subsequently treated with surgical excision of the affected areas, with secondary linear closure. The patient developed keloids at the 2 excision sites and underwent 2 rounds of intralesional triamcinolone injections. Unfortunately, the patient has since been lost to follow-up.

## Discussion

Subcutaneous mercury injection is a rare cause of foreign-body granulomas. It is most commonly associated with magico-religious practices and is seen in the religious practices of Santería, Palo Mayombé, Candomblé, Voodoo, Espiritismo and Yoruba Orisha.^1^^,^^2^ Mercury is also used in Hindu practice as a major constituent of Parad (an alloy of Mercury and Silver or Tin), from which religious relics are made.^2^ The most common route of exposure is through the vaporization of mercury in households as a method to protect and purify the inhabitants.^2^ Acutely, subcutaneous mercury injection can cause localized pain and swelling.^3^, ^4^, ^5^, ^6^ While cutaneous elemental mercury deposits typically cause limited systemic effects, prominent systemic toxicity can rarely occur. While more commonly described with oral ingestion or inhalation of mercury, systemic mercury toxicity findings can vary depending on the type of mercury the patient has been exposed to.^3^ The main mechanism by which mercury causes toxicity is by altering the tertiary and quaternary structure of proteins by binding to sulfhydryl and selenohydryl groups.^3^ Exposure to inhaled mercury vapor can induce pneumonitis, and commonly can cause brain toxicity, leading to neurologic and psychologic complications.^3^ Oral ingestion of mercuric salts and cause GI toxicity and can ultimately lead to shock and acute renal failure.^3^ Exposure to toxic amounts of organic mercury (such as methyl mercury and ethyl mercury), which are usually ingested in contaminated foods, can lead to profound neurologic complications, including cerebral palsy if exposed prenatally.^3^ Interestingly, blood and urine levels indicate mercury exposure but do not correlate well with toxicity.^3^

On histopathology, mercury is characterized as dark, opaque, and spherical globules.^4^, ^5^, ^6^, ^7^, ^8^, ^9^, ^10^ Early localized cutaneous reactions can show acute inflammation, skin necrosis, and abscess formation on histopathology.^4^^,^^7^ Late reactions (as in our case) is typically characterized by granuloma formation composed of foreign-body type giant cells around mercury deposits with a mixed inflammatory infiltrate composed of neutrophils, lymphocytes, histiocytes cells, and, sometimes, plasma cells and eosinophils.^5^^,^^6^^,^^8^^,^^9^ When injected deep enough, it can lead to panniculitis.^10^

We, herein report a case of cutaneous mercury induced granuloma secondary to intentional injection. The difficulty in diagnosis was likely, in some part, due to the patient’s initial reluctance to admit to the practice. Given this practice may be more prevalent than is acknowledged by patients, clinicians should be aware and sensitive to particular cultural practices which may be associated with exposure.

## Conflicts of interest

None disclosed.
